# The Effect of Shot Peening on Corrosion Resistance of 18Ni300 Maraging Steel Manufactured by LPBF

**DOI:** 10.3390/ma19081619

**Published:** 2026-04-17

**Authors:** Ji-Min Yun, Ho-Seok Nam, Ki-Hang Shin, Kwon-Hoo Kim, Ki-Woo Nam

**Affiliations:** 1 Department of Marine Design Convergence Engineering, Pukyong National University, Busan 48513, Republic of Korea; dbswlals333@naver.com (J.-M.Y.); mrppeng@pknu.ac.kr (K.-H.K.); 2 Busan Development Institue, Busan 47210, Republic of Korea; namhs85@bdi.re.kr; 3 Department of Metallurgical Engineering, Pukyong National University, Busan 48513, Republic of Korea; kiki64kr@posco.com; 4 Department of Materials Science and Engineering, Pukyong National University, Busan 48513, Republic of Korea

**Keywords:** additive manufacturing, corrosion resistance, maraging steel, shot peening

## Abstract

This study investigated the correlation between mechanical strengthening and electrochemical corrosion behavior in 18Ni300 maraging steel fabricated via laser powder bed fusion (LPBF). To evaluate the impact of post-processing, specimens were analyzed under four conditions: solution treated (S), solution peened (SP), solution aged (SA), and solution aged peened (SAP). The aging treatment (490 °C for 6 h) effectively enhanced the corrosion resistance by homogenizing the martensitic matrix and promoting the formation of a stable passive film, resulting in the lowest corrosion current density ($i_{corr}$ of 1.716 × 10^−6^ A/cm^2^). In contrast, the application of shot peening after aging (SAP) significantly degraded the corrosion resistance, characterized by the most negative corrosion potential ($E_{corr}$ of −0.374 V and a 2.4 times increase in $i_{corr}$ compared to the SA condition. Quantitative analysis revealed that the 1250 MPa of compressive residual stress induced by peening increased the thermodynamic instability of the surface through extreme lattice distortion, thereby lowering the activation energy for anodic dissolution. Furthermore, the increased surface roughness (60.68 µm) expanded the effective electrochemical reaction area, acting as a kinetic accelerator for corrosion. The results demonstrate that while the SA process provides an optimal balance between microstructural stability and corrosion resistance, additional shot peening (SAP) imposes a significant corrosion penalty despite its mechanical benefits. This study concludes that for 18Ni300 maraging steel, the trade-off between mechanical reinforcement and electrochemical stability must be carefully managed, emphasizing the need for surface stabilization when high-intensity peening is applied in corrosive environments.

## 1. Introduction

Laser Powder Bed Fusion (LPBF) is a 3D metal printing technique that creates objects from almost any metal alloy by precisely welding powdered metal with a high-power laser [1]. This method utilizes Selective Laser Melting (SLM) equipment, where the laser moves slowly across the surface to sinter a very thin layer of metal powder. This process allows the particles to fuse together without the metal needing to melt completely. Consequently, LPBF builds 3D objects, including porous metal parts, by stacking extremely thin layers.

Recently, this technology has been employed to produce high-quality, near-full-density 3D functional components across various industrial sectors. As LPBF technology advances, ongoing research aims to enhance its effectiveness as a sustainable solution for the digital transformation of metallic materials and to validate new metal materials suitable for digital products. Elaziem et al. [2] have highlighted recent development trends in LPBF technology, focusing on process parameters, defects, microstructural evolution, metallurgical phenomena, and the micro-selective laser melting process for creating miniature components.

LPBF technology presents several advantages over traditional manufacturing methods. It allows for the creation of components with intricate geometries without the need for additional tooling, offering nearly complete geometric freedom. Key features of LPBF include high material utilization, exceptional flexibility, and significantly shorter lead times. Moreover, various high-density, near-net-shape components can be produced in a single build batch [3]. As a result, LPBF has attracted considerable interest for manufacturing a diverse array of metallic components, such as STS [4], Ti [5], A286 steel [6], Inconel [7], Ni [8], and Co-Cr alloys [9].

Maraging steel, a low-carbon martensitic steel, is engineered to provide both high strength and remarkable toughness, largely due to precipitation hardening [10]. Additively manufactured (AM) maraging steel specimens produced at rapid solidification rates are the focus of extensive research, particularly because they exhibit numerous nanocrystals and acicular structures on their surfaces [11].

Allam et al. [12] studied the relationship between nanoprecipitate formation and the development of porous, dendritic microstructures with micro-segregation in Grade 300 maraging steel fabricated via Laser Powder Bed Fusion (LPBF) after aging and solution-aging treatments. Deirmina et al. [13] explored how varying nitrogen (N) and oxygen (O) contents in raw materials affect the fatigue properties of LPBF-processed 18Ni300, considering direct aging, homogenization, and solution annealing followed by aging. Additionally, Tezel and Kovan [14] investigated the rotating bending fatigue behavior of LPBF-fabricated maraging steel under different heat treatment conditions. Vishwakarma et al. [15] examined the low-cycle fatigue behavior of PBF-LB maraging steel across various build orientations and assessed the effects of heat treatment on its fatigue life.

Maraging steels are widely used in industries such as aerospace and tooling [16], and they are currently being actively researched for applications in offshore structures [17]. The mechanical properties of maraging steels surpass those of traditional steels commonly used in the maritime sector, including 17-4 PH, 15-5 PH, and duplex stainless steels [18,19,20]. Most studies on maraging steel have concentrated on assessing the tensile, fracture, and fatigue properties of components produced through the LPBF process [21,22,23,24,25]. Various strategies have been explored to improve fatigue strength, such as altering chemical composition, nitriding [26], and shot peening [27,28,29,30]. It has been reported that surface hardening through nitriding [26] significantly enhances the fatigue limit in conventional maraging steels.

Recent research has also focused on the corrosion resistance of maraging steel. For example, Zhao et al. [31] examined the effect of porosity on the corrosion behavior and mechanical properties of SLM 18Ni300 maraging steel. They found that the porosity fraction decreased after solution treatment, and aging treatment led to a reduction in pore size, resulting in improved corrosion resistance. Tonolini et al. [32] conducted a comparative study on the corrosion behavior of 18Ni300 maraging steel produced via LPBF versus conventional melting methods. In their polarization tests, LPBF specimens demonstrated superior corrosion resistance, exhibiting a higher corrosion potential and lower corrosion current density compared to melted specimens. Lek et al. [33] performed polarization tests on LDED-fabricated maraging steel M789 specimens subjected to direct aging and solution treatment followed by aging. Their findings indicated that, compared to 15-5 PH steel made via LDED and LPBF, M789 specimens showed greater susceptibility to pitting corrosion in both the as-built and solution-treated conditions, with as-built specimens being more prone to pitting due to the presence of austenite and the absence of precipitates. Additionally, intrinsic differences in manufacturing characteristics between LPBF and LDED can lead to variations in the microstructure and mechanical properties of 3D fabricated parts [34,35].

Several studies have reported that while lattice structure evolution can improve thermal and mechanical properties, it may also compromise corrosion resistance. This phenomenon is similar to the way shot peening enhances mechanical characteristics through the creation of compressive residual stress via lattice distortion, often resulting in reduced corrosion resistance. Abdelfatah et al. [36] showed that preheating in dissimilar friction stir welding (FSW) of AA2024 (base metal) and AA7075 (reinforcement) enhances the stability of the passive film, thus improving corrosion protection. Okail et al. [37] examined FSW with an AA7075 interlayer between AA2024 base metals, demonstrating that while the joint is prone to corrosion due to the galvanic potential difference in the stir zone, incorporating an interlayer combined with optimized post-weld heat treatment (PWHT) is crucial for maximizing overall performance. Furthermore, Oqail et al. [38] established that Y_2_O_3_ particles significantly enhance mechanical strength and wear resistance of composites through dispersion strengthening, confirming that, within optimal concentration limits, Y_2_O_3_ is a highly effective additive for extending the service life of electrical brushes.

In this study, we comprehensively investigated the corrosion resistance of 18Ni300 maraging steel fabricated via Laser Powder Bed Fusion (LPBF). Specifically, we systematically analyzed and compared the microstructural evolution, mechanical properties, and electrochemical corrosion behavior of three distinct conditions: solution treated (S), solution aged (SA), and shot peened (SP) specimens.

## 2. Materials and Experimental Methods

**Figure 1:**
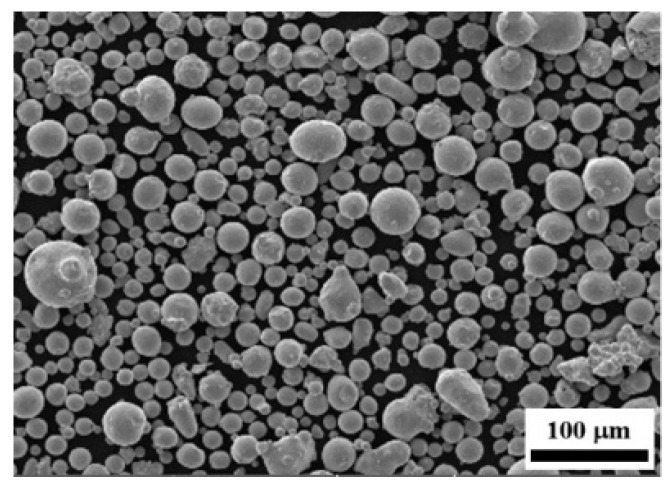
SEM micrograph of maraging steel powder with inclusions on the surface of some particles.

The study utilized EOS Maraging Steel MS1 particles (Turku, Finland), which are gas-atomized powders with a particle size of less than 63 µm. Table 1 and Figure 1 present the chemical composition of the powder as detailed in the supplier’s datasheet, along with the powder morphology observed using scanning electron microscopy (SEM; VEGA3 TESCAN, Brno, Czech Republic). The particles are predominantly spherical, with some showing surface deposits. These deposits form when smaller droplets adhere to the surfaces of larger particles during the gas atomization process.

**Figure 2:**
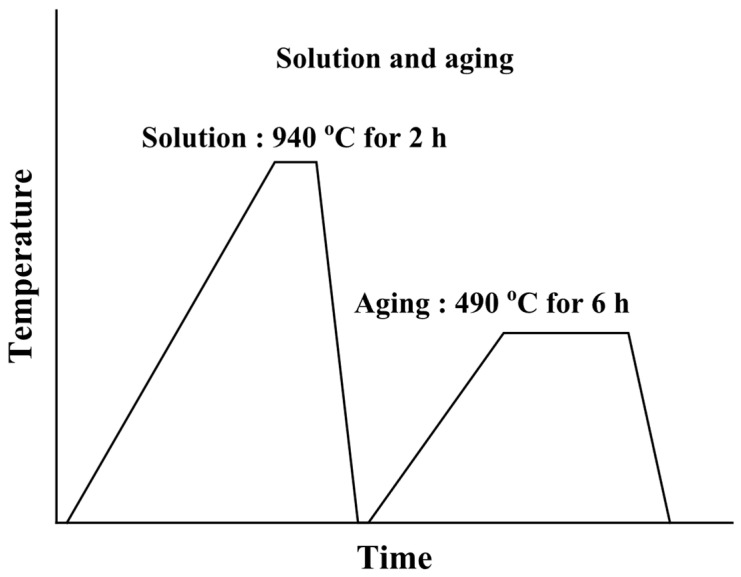
Schematic illustration of the heat treatment method employed in this work.

3D additive manufacturing was performed using an EOS M290 system (Munich, Germany) equipped with an ytterbium fiber laser. This laser has a maximum power of 400 W, a beam spot diameter of approximately 0.1 mm, a wavelength of 1.06–1.10 µm, and a maximum scanning speed of 7 m/s. The laser power was kept between 370 and 400 W, with a layer thickness of 40 µm and a beam spot diameter of 100 µm. Quality assurance testing and preparation of analytical samples adhered to ASTM B215 standards [39]. The specimens measured 16 mm in diameter and 180 mm in length. Figure 2 illustrates the heat treatment method used in this study.

Heat treatment was conducted in a box furnace (Elite Thermal Systems Limited, BRF 14/5–2416, Leicestershire, England). The additive-manufactured maraging steel underwent solution treatment at 940 °C for 2 h, followed by air cooling, and was then aged at 490 °C for 6, again followed by air cooling. Any residual stresses on the specimen surface resulting from heat treatment were removed by polishing with #2000 emery paper. Four types of specimens were utilized: solution treated (S), solution peened (SP), solution aged (SA), and solution aged peened (SAP). Additionally, four types of specimens were prepared for polarization tests to evaluate the effects of shot peening. The conditions for SP was conducted using air pressure of 0.64 MPa and time of 8 s. Surface roughness was measured the centerline average roughness (Ra) using a Laser Microscope (VK-X1050, Keyence, Osaka, Japan) [40].

The polished and etched vertical cross-sections of the 3D additive-manufactured specimens, oriented relative to the build direction, were examined using optical microscopy (GX51F, Olympus, Tokyo, Japan) and field-emission scanning electron microscopy (FE-SEM, Zeiss Sigma560, Germany) for microstructural analysis. Vickers hardness was measured from the surface into the depth direction with an indentation load of 4.9 N and a dwell time of 10 s.

Mechanical properties were assessed through tensile testing using a universal testing machine (UTM; Shimadzu UH-F100A, Japan, 98 kN capacity) in accordance with ASTM E8 standards [41]. The crosshead speed was maintained at 0.5 mm/min during the tests. The fracture morphology of the specimens produced by Direct Metal Laser Sintering (DMLS) was further analyzed using FE-SEM.

Potentiodynamic polarization tests were performed on mirror-polished specimens measuring 10 mm × 10 mm using a VersaSTAT 3 potentiostat (AMETEK, Berwyn, PA, USA) in a 3.5 wt% NaCl solution at room temperature. The setup employed a three-electrode system, with the specimen serving as the working electrode (WE), a platinum sheet (20 mm × 20 mm) as the counter electrode (CE), and a saturated KCl Ag/AgCl electrode as the reference electrode. Potentiodynamic polarization curves were recorded across a voltage range of −500 to +500 mV at a scan rate of 20 mV/min.

Tensile, hardness, and polarization tests were conducted on three specimens of each type, and the average values were used.

For the immersion tests, mirror-polished specimens (10 mm × 10 mm) were submerged in a 3.5 wt% NaCl solution at room temperature. The surface morphology was observed using SEM at intervals of 1, 5, 10, and 120 h, followed by EDX line profile analysis to evaluate the progression of corrosion. The immersion test used three specimens of each time.

## 3. Results and Discussion

**Figure 3:**
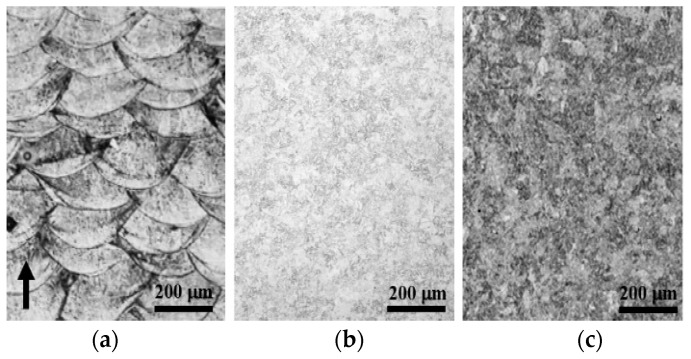
Vertical cross-sectional optical micrographs. (**a**) as-built specimen, (**b**) S specimen, (**c**) SA specimen.

Figure 3a–c illustrate the microstructures of the as-built, solution treated (S), and solution aged (SA) specimens as observed through optical microscopy. In Figure 3a, the as-built specimen exhibits a fish-scale morphology, characterized by boundaries arranged in alternating layers. These boundaries were formed by the scanning pattern, which involves a 90° rotation between each adjacent layer. In contrast, the fish-scale-like melt pool boundaries are absent in the S (Figure 3b) and SA (Figure 3c) specimens.

**Figure 4:**
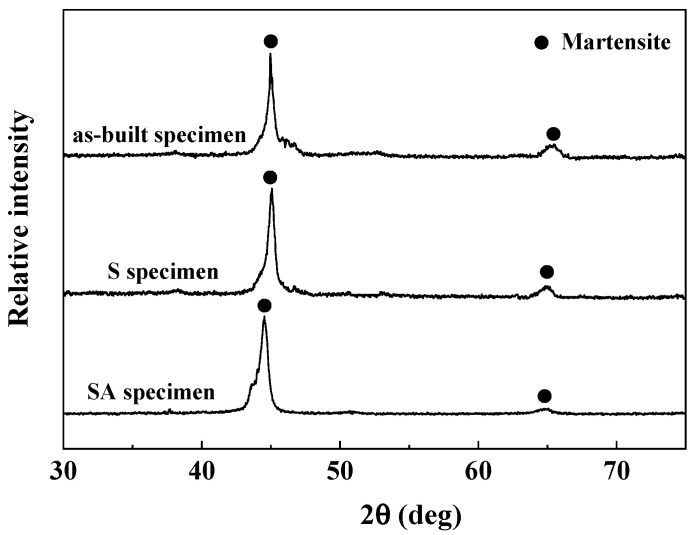
XRD profiles of as-built, S specimen and SA specimen of maraging steel.

Figure 4 presents the XRD (X-ray diffraction) results for the as-built, S, and SA specimens. All three specimen types exhibited diffraction peaks corresponding to the (110) and (200) planes, indicating a predominant martensitic phase.

Regarding phase stability, although aging can theoretically induce martensite reversion in maraging steels, the significant increase in hardness observed in the SA specimen suggests that precipitation hardening by Ni_3_(Ti, Mo) is the primary mechanism at play under the current aging conditions (490 °C for 6 h). The lack of a decrease in mechanical strength indicates that the formation of reverted austenite was insufficient to compromise the martensitic matrix. Future studies utilizing Rietveld refinement of XRD data or EBSD analysis will enable a more accurate quantification of any minor austenite fractions.

**Figure 5:**
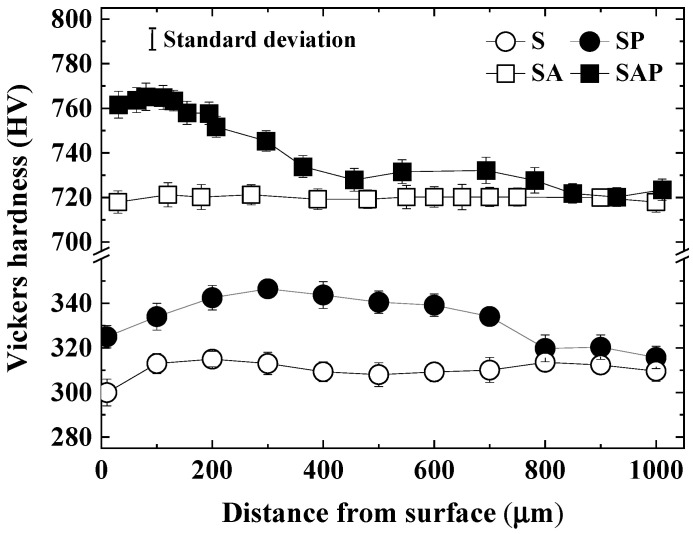
Vickers hardness of S, SP, SA and SAP specimens.

Figure 5 displays the Vickers hardness values for the four types of specimens, with three measurements taken for each type. The solid line in the figure represents the standard deviation. The hardness of the S specimen ranged from 300 to 315 HV, while the hardness of the SA specimen significantly increased to 718–721 HV. For the S specimen, hardness measured 300 HV at the surface and 315 HV at a depth of 200 µm, yielding a consistent average of 307 HV. In contrast, the average hardness of the SA specimen remained steady at approximately 720 HV. Additionally, the hardness of the SP specimen ranged from 325 to 347 HV, indicating an improvement compared to the S specimen.

The SAP showed a surface hardness of approximately 762 HV, reaching 765 HV at a depth of 100 µm. After that, the HV of SAP gradually decreased, and was similar to the HV of SA at approximately 800 µm. The hardness increased significantly up to a depth of 300 µm due to the shot peening effect. Previous studies [42,43,44] indicate that steels with a cellular structure smaller than 1 µm exhibit superior strength and hardness. Additionally, the hardness of the additive-manufactured maraging steel increased significantly after heat treatment. Huang et al. [45] reported that in the LPBF process, the fine structure caused by rapid solidification and the surface refinement caused by shot peening influenced the improvement of hardness. Aging is a standard process for maraging steels characterized by a low-carbon body-centered cubic (BCC) martensitic structure. During martensitic aging, fine intermetallic precipitates rich in Ni, Co, and Mo are uniformly distributed, which strengthens the martensitic matrix [46]. Moreover, shot peening induces plastic deformation in the martensitic structure, leading to work hardening and an additional increase in hardness [45]. Consequently, precipitation hardening is the primary strengthening mechanism in maraging steel. In addition, by the shot peening, the surface compressive residual stresses were measured as follows: 2 MPa for the S specimen, 807 MPa for the SP specimen, 200 MPa for the SA specimen, and 1250 MPa for the SAP specimen. Additionally, the surface roughness (Ra) values were 59.03 µm for the S specimen, 56.57 µm for the SP specimen, 56.29 µm for the SA specimen, and 60.68 µm for the SAP specimen.

**Figure 6:**
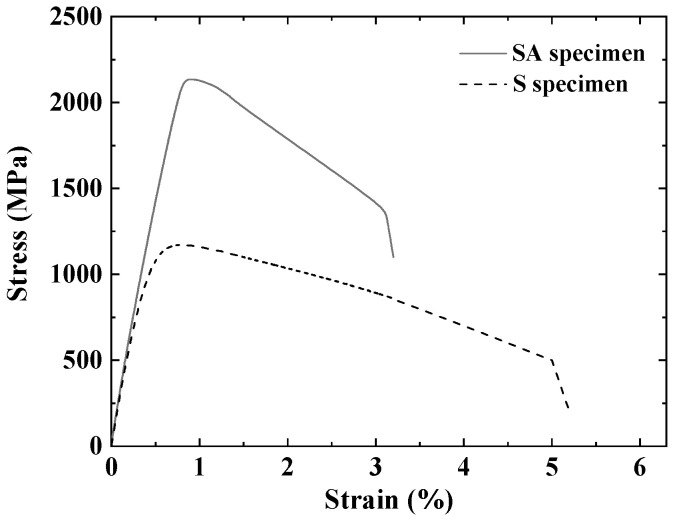
Stress–strain curves of S specimen and SA specimen.

Figure 6 presents the representative stress–strain curves for uniaxial tensile specimens made from 3D additive-manufactured maraging steel. Tensile tests were performed on both the S and SA specimens, and the measured mechanical properties are summarized in Table 2. Table 2 shows the mean and standard deviation. The S specimen demonstrated an ultimate tensile strength ($\sigma_{t}$) of 1170 MPa and a yield strength ($\sigma_{y}$) of 933 MPa. In contrast, the SA specimen exhibited significantly higher values of 2134 MPa($\sigma_{t}$) and 2059 MPa($\sigma_{y}$), indicating an approximately 82% increase in tensile strength compared to the S specimen.

Aging significantly improves the tensile strength of additive-manufactured maraging steel. Kempen et al. [43] attributed this enhancement to the formation of specific alloying elements, such as Ni, Mo, and Ti, in the microstructure during heat treatment. Zhao et al. [47] discovered that the presence of Ni_3_Mo particles within the martensite strengthens the material through mechanisms like dislocation accumulation, stress concentration, and increased micro-strength after heat treatment. Unlike carbides, the intermetallic compound Ni_3_(Ti, Mo) serves primarily as an obstacle, limiting slip distance rather than merely restricting dislocation movement [48,49,50]. On the other hand, the elongation (ε) at break decreased from 5.1% to 3.1% after aging. This indicates that while the strength of aged maraging steel is significantly increased, its ductility is correspondingly decreased.

**Figure 7:**
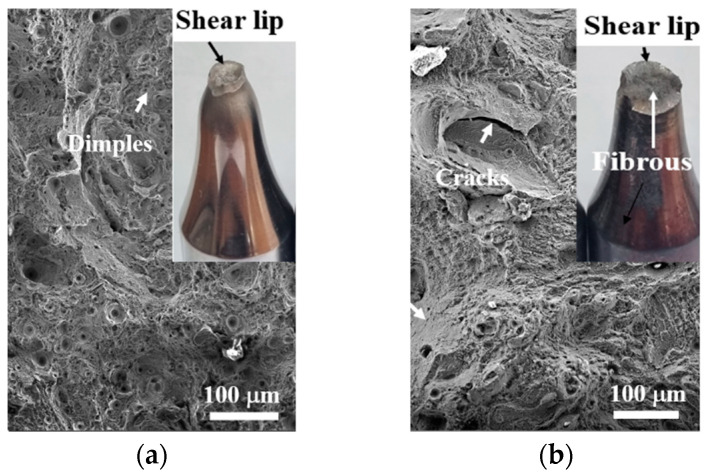
SEM images of fracture surfaces. (**a**) S specimen, (**b**) SA specimen.

Figure 7 shows the observation results of the fracture morphology of the tensile specimens. Panels (a) and (b) correspond to the S specimen and the SA specimen, respectively. The fracture of the S specimen, which showed a prominent shear lip and considerable necking, was linked to a ductile fracture mechanism involving the nucleation, growth, and coalescence of voids. In contrast, the fracture surface of the SA specimen had little to no shear lip, and its central region displayed quasi-cleavage characteristics, evident in its fibrous fracture surface. Bai et al. [51] noted that large pores mainly arise from defects like unmelted powder and keyhole pores. Furthermore, some pores that form near the crack tip can accelerate crack propagation and diminish plasticity.

**Figure 8:**
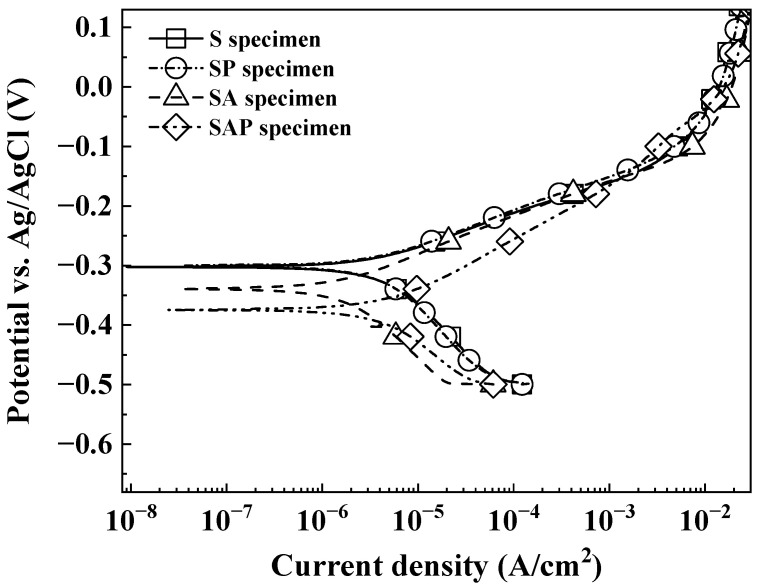
Potentiodynamic polarization curves of S, SP, SA and SAP specimens.

Figure 8 shows the results of displays the polarization curve measurements for four specimen types (S, SP, SA, and SAP) in a 3.5 wt% NaCl solution at room temperature. Maraging steel, known for its martensitic structure and high hardness, can benefit from surface modifications like shot peening to enhance surface hardness and boost the fatigue limit. This study evaluated the polarization characteristics by applying shot peening to both solution-treated and aged specimens. To ensure reproducibility, each electrochemical measurement was conducted at least three times under the same conditions. The results in Table 3 are presented as mean values. The standard deviation for $E_{corr}$ ranged from ±5 mV to ±25 mV, while the $i_{corr}$ values exhibited a standard deviation of approximately 10~15%, which is within the statistically acceptable range for potentiodynamic polarization tests in a 3.5 wt% NaCl solution.

The corrosion potential ($E_{corr}$) is a thermodynamic indicator representing the ‘propensity’ of a material to undergo corrosion. In general, as the $E_{corr}$ value shifts in a more negative direction, the material becomes chemically more active, implying a state where corrosion is more likely to initiate. The SAP specimen exhibited a value of −0.374 V, making it the most negatively charged among the four specimen types. This indicates that the SAP specimen is the most thermodynamically unstable and is situated in an environment conducive to corrosion initiation. The practical corrosion resistance, or corrosion rate, is primarily determined by the corrosion current density ($i_{corr}$). The SA specimen exhibited the lowest $i_{corr}$ of 1.716 × 10^−6^ A/cm^2^, indicating that the microstructure evolved through aging treatment effectively inhibited the corrosion rate. In contrast, the SAP specimen recorded the highest $i_{corr}$ of 4.198 × 10^−6^ A/cm^2^. This shows that the shot peening conducted after aging increased the corrosion rate, likely due to heightened surface roughness and induced lattice distortion. Notably, the SAP specimen displayed both the most negative corrosion potential (−0.374 V) and the highest corrosion current density (4.198 × 10^−6^ A/cm^2^). This demonstrates that while shot peening can improve the mechanical strength of maraging steel, it also contributes to corrosion initiation and accelerates the dissolution rate, ultimately diminishing overall electrochemical stability. As a result, the SA specimen exhibited superior corrosion resistance, whereas the SAP specimen, which underwent additional peening, showed the poorest corrosion behavior. Notably, the three types of specimens (excluding SAP) had lower corrosion rates compared to the LPBF-fabricated 18Ni300 maraging steel reported by Suryawanshi et al. [52], and the SA specimen displayed a slower corrosion rate than those reported by Tonolini et al. [32].

The anodic Tafel slope ($\beta_{a}$) represents the resistance to metal ionization. For the SAP specimen, the slope becomes more gradual and shifts to the right, indicating that the high dislocation density induced by shot peening has reduced the activation energy needed for anodic dissolution. The cathodic Tafel slope ($\beta_{c}$) indicates the rate of the oxygen reduction reaction. While similar slopes were observed across all specimens, the SAP specimen exhibited an increased limiting diffusion current density due to its higher surface roughness, resulting in the intersection point ($i_{corr}$) forming at a significantly higher value. The SA specimen (1.716 × 10^−6^ A/cm^2^) exhibited the lowest $i_{corr}$ because the aging treatment homogenized the precipitates, stabilizing the corrosion initiation sites. In contrast, the $i_{corr}$ of the SAP specimen (4.198 × 10^−6^ A/cm^2^) surged approximately 2.4 times compared to the SA specimen. This shows that the increase in chemical activity, caused by lattice distortion and heightened surface roughness, dominated the corrosion mechanism, outweighing the potential benefits of the compressive residual stress introduced by peening.

Therefore, the corrosion resistance of LPBF-fabricated 18Ni300 maraging steel was significantly affected by post-processing treatments. As shown in Figure 8 and Table 3, the SA specimen exhibited the lowest corrosion current density ($i_{corr}$ = 1.716 × 10^−6^ A/cm^2^), indicating the superior stability of its microstructure after aging treatment. In contrast, the SAP specimen showed the highest $i_{corr}$ (4.198 × 10^−6^ A/cm^2^), representing a 144% increase in corrosion rate compared to the SA specimen condition. A critical observation is the negative shift in the corrosion potential ($E_{corr}$) in the SAP specimen (−0.374 V). In thermodynamic terms, a more negative value indicates higher chemical activity and an increased likelihood of corrosion initiation. The accelerated corrosion in SAP is due to the synergistic effect of high-density dislocations and lattice distortion caused by shot peening. These crystalline defects serve as high-energy sites that reduce the activation energy required for anodic dissolution. Furthermore, the increased surface roughness from peening facilitates oxygen reduction reactions on the cathode, thereby increasing the overall $i_{corr}$. Therefore, while shot peening enhances the mechanical strength of maraging steel, it negatively impacts its electrochemical stability in chloride-rich environments.

**Figure 9:**
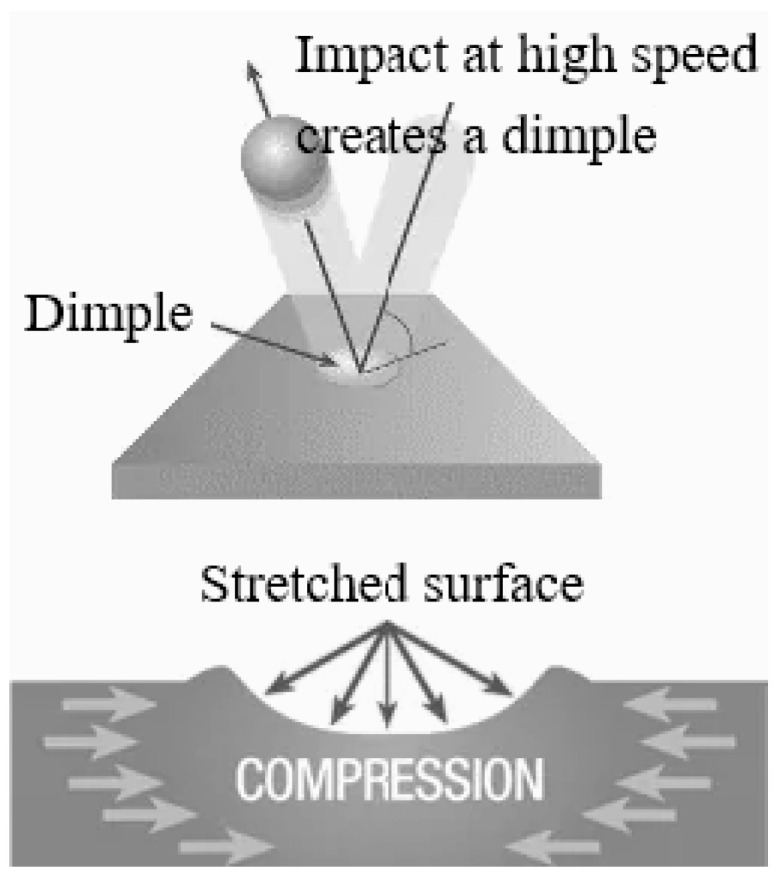
Schematic diagram of compressive residual stress generating by shot peening.

The shot peening process occurs when high-velocity shots collide with a metal surface, creating microscopic dimples at the points of impact, as shown in Figure 9. During this process, the impact displaces the metal grains in the surface layer, causing plastic deformation (permanent deformation). Meanwhile, the sub-surface layers, which do not experience direct impact, retain their original shape due to their elastic properties. As a result, a force is generated as the inner layers pull the surface layer inward, inducing compressive residual stress in the surface layer where the metal grains are tightly pressed together. While the compressive residual stresses from shot peening enhance fatigue life [53], they also increase surface roughness, which can act as a corrosion initiation site under certain conditions [54].

**Figure 10:**
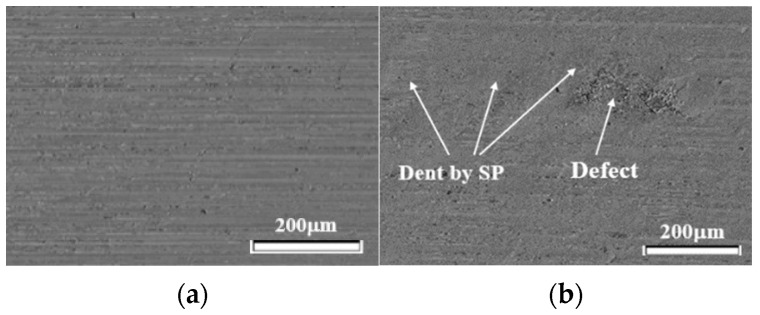
Surfaces observation before and after SP on SAP specimen. (**a**) Before, (**b**) After.

Figure 10 shows the surface before and after SP on SAP specimen. (a) is before SP, and (b) is after SP. (a) shows no defects on the surface, while (b) shows defects.

The electrochemical corrosion behavior of LPBF-fabricated 18Ni300 maraging steel is influenced by a complex interplay of microstructural evolution (precipitation), surface topography, and the energetic state of the lattice. The superior corrosion resistance of the SA specimen ($i_{corr}$ = 1.716 × 10^−6^ A/cm^2^) is primarily attributed to the homogenization of the microstructure through aging. The uniform precipitation of intermetallic compounds, such as Ni_3_(Ti, Mo), reduces chemical micro-segregation within the martensitic matrix. This uniformity encourages the development of a more continuous and defect-free passive film, serving as a strong barrier against chloride ion (Cl^−^) penetration. In contrast, the S specimen retains inherent porosity from the LPBF process, while the SA specimen stabilizes the areas around these pores, reducing the formation of localized galvanic cells. A key finding of this study is the significant reduction in corrosion resistance observed in the SAP specimen, which displayed the most negative corrosion potential ($E_{corr}$ = −0.374 V). This degradation can be quantitatively correlated with compressive residual stress. The SAP specimen exhibits an exceptionally high compressive residual stress of 1250 MPa, which is 6.25 times greater than that of the SA specimen (200 MPa). This intense stress level indicates a state of extreme lattice distortion and high dislocation density. From a thermodynamic perspective, these structural defects increase the Gibbs free energy of the surface atoms, which effectively lowers the activation energy required for anodic dissolution (Fe → Fe^2+^ + 2e^−^). Consequently, the SAP specimen becomes thermodynamically more active, leading to earlier corrosion initiation and a more negative $E_{corr}$ compared to other conditions. The acceleration of the corrosion rate in the SAP specimen is further quantified by the surface roughness data. The SAP specimen exhibited the highest surface roughness of 60.68 µm (compared to 56.29 µm for SA specimen). Although the numerical increase in roughness may seem moderate, the shot peening process produces a distinct morphology characterized by overlapping dimples. These micro-indentations significantly enhance the effective electrochemical surface area in contact with the electrolyte, providing more active sites for the cathodic oxygen reduction reaction (O_2_ + 2H_2_O + 4e^−^ → 4OH^−^). The synergy between the lowered anodic activation energy (from the 1250 MPa stress) and the increased cathodic reaction area (from the 60.68 µm roughness led to a 2.4 times increase in $i_{corr}$ for the SAP specimen compared to the SA specimen. Therefore, while the SAP specimen is highly effective for mechanical strengthening but comes with a significant ‘corrosion penalty’. The 1250 MPa of residual stress promotes initiation, while the increased surface roughness accelerates the dissolution rate. For corrosive environments, the SA specimen offers the best balance, whereas the SAP specimen requires additional surface stabilization to mitigate its harmful electrochemical effects. Although Electrochemical Impedance Spectroscopy (EIS) was not used in this study to directly measure polarization resistance, the stability of the passive film can be inferred from the anodic polarization behavior and corrosion current density ($i_{corr}$). The SA specimen showed the lowest $i_{corr}$ (1.716 × 10^−6^ A/cm^2^), which suggests the formation of a more compact and defect-free passive layer. This stability is likely a result of microstructural homogenization during aging, which reduces the number of active sites for pit initiation. In contrast, the SAP specimen, despite having a high compressive residual stress (1250 MPa), exhibited a higher $i_{corr}$ and a more negative $E_{corr}$. This suggests that the intense lattice distortion and increased surface roughness (60.68 µm) impede the formation of a stable passive film. As a result, the surface remains in an ‘active’ state, where the rate of metal dissolution surpasses the rate of film repair, causing the degradation in corrosion resistance that we observe.

**Figure 11:**
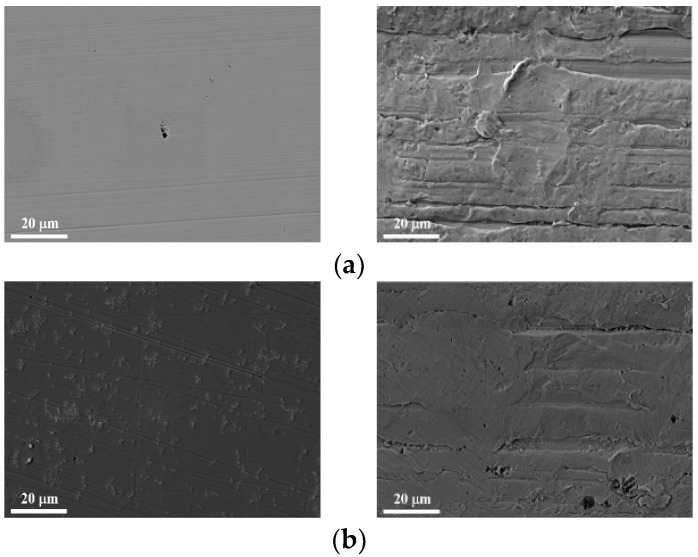

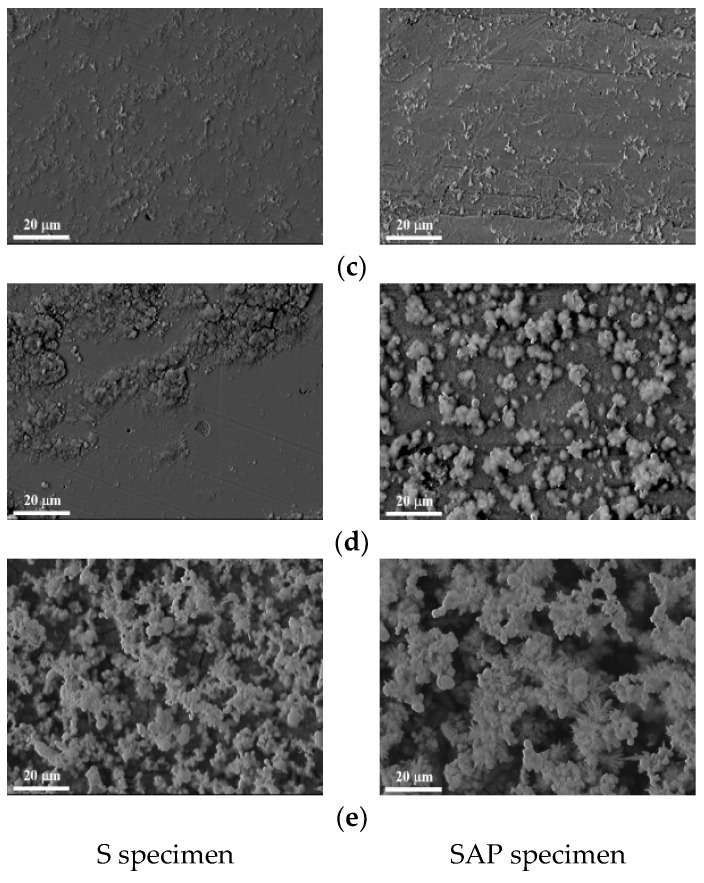
Surface morphology of S and SAP specimens according to immersion time. (**a**) 0, (**b**) 1, (**c**) 5, (**d**) 10, (**e**) 120.

The surface morphologies of four specimen types (S, SP, SA, and SAP) were examined using SEM after immersion in a 3.5 wt% NaCl solution at room temperature for durations ranging from 0 to 120 h. Figure 11 presents the results for the S and SAP specimens. In the 0 h specimens shown in panel (a), pores and linear defects are visible on the surface. Previous studies have indicated that the additive manufacturing method used in this research is prone to keyhole porosity, which is highly sensitive to the scanning strategy and deposition direction [55]. The SAP specimen also displays linear defects caused by aging and dimples resulting from shot peening. Panels (b), (c), and (d) illustrate the surfaces of the specimens after 1, 5, and 10 h of immersion, respectively, and show a clear increase in the accumulation of corrosion deposits with longer immersion times. The SP specimen exhibited a surface condition similar to that of the S specimen, suggesting the formation of a comparable protective film (see Figure 8).

It has been reported that the shift in the $E_{corr}$ value toward a more negative potential is associated with the formation of a protective film covering the entire surface of the specimen [47]. After the immersion test, as shown in (b) and (c), the S specimen exhibited a homogeneous and uniform protective film on its surface. linked to the formation of a protective film covering the entire surface of the specimen [45]. After the immersion test, as shown in (b) and (c), the S specimen displayed a homogeneous and uniform protective film on its surface.

Furthermore, no cracks were observed in this passive barrier. In contrast, the shift in the $E_{corr}$ value toward a more negative potential in the SA specimen is linked to the formation of a non-protective film that covers the entire surface. However, due to the increase in strength, the $i_{corr}$ value was the lowest, making it difficult for the electrolyte to penetrate the film. In contrast, the SAP specimen exhibited rapid corrosion due to the defects shown in Figure 10, which facilitated the penetration of the electrolyte through the film. Panel (e) displays the specimen after the 120 h immersion test, revealing that the surface has corroded and the deposited metal is exposed.

While the immersion test results were primarily analyzed through qualitative surface observations, these observations are quantitatively supported by the corrosion current density ($i_{corr}$) derived from the potentiodynamic polarization tests. According to Faraday’s law, $i_{corr}$ is directly proportional to the mass loss rate. The SAP specimen, which exhibited the highest $i_{corr}$ (4.198 × 10^−6^ A/cm^2^), showed the most severe surface degradation (e.g., deep pitting and oxide scale formation) during immersion. This qualitative degradation aligns with the 1250 MPa of compressive residual stress and the 60.68 mm of surface roughness, which together accelerated the dissolution kinetics compared to the SA specimen, which retained a relatively intact surface.

**Figure 12:**
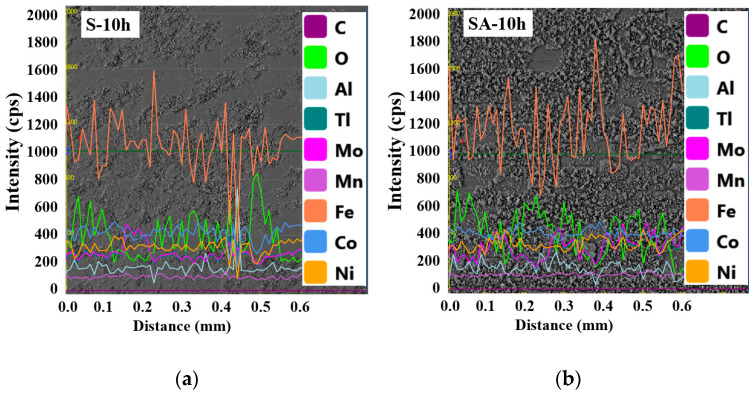
Line profile after 10 h of immersion. (**a**) S specimen, (**b**) SA specimen.

Figure 12 shows the line profile of the surface after 10 h of immersion. Panels (a) and (b) display the S specimen and the SA specimen, respectively. A significant amount of corrosion deposits is evident on the surfaces of both specimens, characterized by a high concentration of oxygen (O). Thus, the corrosion deposits can be identified as oxides.

**Figure 13:**
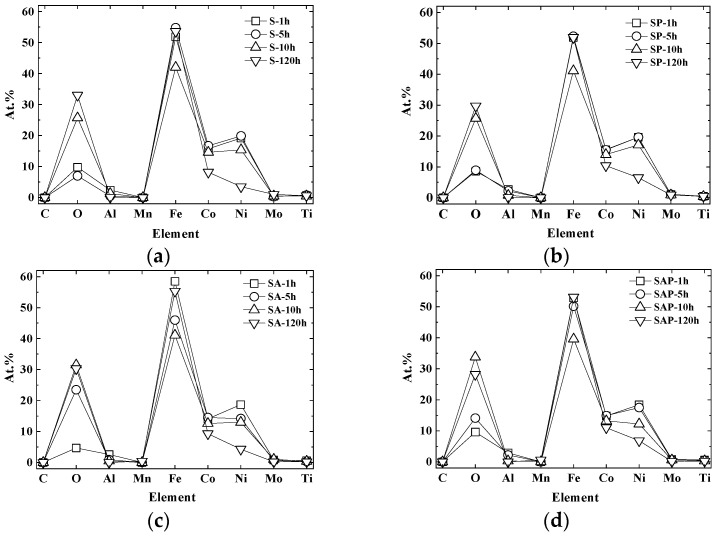
Distribution of each chemical component according to immersion time. (**a**) S specimen, (**b**) SP specimen, (**c**) SA specimen, (**d**) SAP specimen.

Figure 13 presents a quantitative analysis of the surface chemical compositions for four types of specimens categorized by immersion time: (a) S specimen, (b) SP specimen, (c) SA specimen, and (d) SAP specimen. The data clearly indicate that the oxygen content increases with immersion time across all specimens. Corrosion begins as the oxygen on the metal surface is consumed, prompting the cathodic reaction to shift to the surface. Simultaneously, chloride ions, which are anions, migrate toward the anode due to the presence of cations. This migration enhances the anodic reaction and increases acidity, thereby accelerating the corrosion process [56]. Over time, the accumulation of corrosion deposits (oxides) on the surface intensifies, allowing corrosion to propagate further through the crevices within these deposits [57].

## 4. Conclusions

In this study, we systematically investigated the effects of post-processing—specifically aging treatment and shot peening—on the mechanical properties and electrochemical corrosion behavior of LPBF-fabricated 18Ni300 maraging steel. The results obtained were as follows:

1. The SA (Solution-Aged) specimen showed the most superior corrosion resistance ($i_{corr}$ = 1.716 × 10^−6^ A/cm^2^). This is attributed to the aging-induced formation of uniform Ni_3_(Ti, Mo) nanoprecipitates, which homogenizes the martensitic matrix. Such microstructural uniformity promotes the formation of a dense and stable passive film, which effectively reduces localized galvanic corrosion sites.
2. The SAP (Solution Aged Peened) specimen demonstrated a significant degradation in corrosion potential ($E_{corr}$ = −0.374 V), the most negative among all conditions. This phenomenon is quantitatively linked to a significant compressive residual stress of 1250 MPa. The substantial accumulation of elastic energy results in extreme lattice distortion and an increased dislocation density, which lowers the activation energy necessary for anodic dissolution (Fe → Fe^2+^ + 2e^−^). As a result, the SAP surface becomes thermodynamically more active, which facilitates earlier corrosion initiation.
3. The 2.4 times increase in $i_{corr}$ for the SAP specimen compared to the SA specimen is driven by the increased surface roughness (60.68 µm). The micro-dimples generated by shot peening expand the effective electrochemical surface area, providing more active sites for the cathodic oxygen reduction reaction (O_2_ + 2H_2_O + 4e^−^ → 4OH^−^). This increase in reactive area serves as a kinetic accelerator, significantly enhancing the overall corrosion current density.
4. The results demonstrate a clear trade-off in 18Ni300 maraging steel. While shot peening (SAP) enhances mechanical strength and fatigue life by creating high compressive stress, it also significantly increases susceptibility to corrosion due to energetic activation and area expansion. For structural applications in corrosive environments, the SA condition offers the best balance. In contrast, the SAP condition requires additional surface stabilization or protective coatings to mitigate its harmful electrochemical effects.

## Figures and Tables

**Table 1:** Chemical composition of maraging steel powder(wt%).

Fe	Ni	Co	Mo	Ti	Cu	Cr	Al	Mn	Si	C	P	S
Bal.	17.56	8.59	4.72	0.78	0.03	0.19	0.11	0.06	0.04	0.02	0.01	0.01

**Table 2:** Mechanical properties.

Specimen	$\sigma_{t}$ (MPa)	$\sigma_{y}$ (MPa)	ε (%)
S	1170 ± 22	933 ± 31	5.1 ± 1.8
SA	2134 ± 15	2059 ± 26	3.1 ± 1.2

**Table 3:** Corrosion potential ($E_{corr}$) and corrosion current density ($i_{corr}$) of 4 types of specimen.

Specimen	Corrosion Potential ($E_{corr}$, V)	Corrosion Current Density ($i_{corr}$, ×10^−6^ A/cm^2^)	Remarks
S specimen	−0.296 ± 0.008	3.075 ± 0.21	Base
SP specimen	−0.300 ± 0.012	3.105 ± 0.35	Similar to S specimen
SA specimen	−0.339 ± 0.010	1.716 ± 0.18	lowest corrosion rate
SAP specimen	−0.374 ± 0.022	4.198 ± 0.52	fastest corrosion rate

## Data Availability

The original contributions presented in this study are included in the article. Further inquiries can be directed to the corresponding author.
